# Musculoskeletal pain and school bag use: a cross-sectional study among Ugandan pupils

**DOI:** 10.1186/1756-0500-7-222

**Published:** 2014-04-09

**Authors:** Erisa S Mwaka, Ian G Munabi, William Buwembo, John Kukkiriza, Joseph Ochieng

**Affiliations:** 1 Anatomy Department, School of Biomedical Sciences, College of Health Sciences, Makerere University, P O Box 7072, Kampala, Uganda

**Keywords:** Musculoskeletal pain, Low back pain, Schoolbag weight, Pupils

## Abstract

**Background:**

Though seen as a convenient method of carrying books and other scholastic materials including food items, schoolbags are believed to contribute to back and other musculoskeletal problems in school going children. This study set out to determine the prevalence of low back and other musculoskeletal pains and describe their relationship with schoolbag use in pupils.

**Results:**

This was a cross-sectional descriptive study involving 532 pupils from six primary schools with a mean age of 13.6 years. Analyses included the chi- square test, independent t tests, regression analysis and test for trend across ordered groups.

Backpacks were the most common type of schoolbag and younger children carried disproportionately heavier bags. Urban pupils were younger, carried significantly heavier bags, and less likely to complain about schoolbag weight than the rural pupils,

About 30.8% of the pupils carried schoolbags which were more than 10% of their body weight. About 88.2% of pupils reported having body pain especially in the neck, shoulders and upper back. About 35.4% of the children reported that carrying the schoolbag was the cause of their musculoskeletal pain. The prevalence of lower back pain was 37.8%. There was significant association between low back pain and; method of bag carriage (p < 0.0001), long duration of walking (odds ratio 2.67, 95% CI 1.38- 5.16) and the time spent sitting after school (p = 0.02). Only 19% had lockers at school.

**Conclusion:**

Urban pupils were younger, carried significantly heavier bags, and less likely to complain about schoolbag weight than the rural pupils. The majority of pupils complained of musculoskeletal pain of which 35.4% was attributed to the schoolbags.

The prevalence of lower back pain was 37.8%. Schools need to provide lockers and functional libraries in order to avoid excessive loading and repetitive strain injuries.

## Background

All over the world there has been an outcry by parents, school officials, and health professionals concerning the carrying of backpack loads beyond the recommended safe load limits of 10% to 15% of body weight by school going children [1,2].

Research in this area shows that although the average loads vary greatly between studies, the majority of reports indicate that the loads carried by students are greater than the recommended limits [3-6]. Some researchers hypothesize that use of heavy backpacks may contribute to the high reports of back pain in children [3,4]. Whereas the overall lifetime prevalence of low back pain (LBP) in children has been reported as high as 65% [7], it ranges between 30% to 50% by other authors [8-12]. This hypothesis of excessive school bag weight leading to LBP forms the basis for the recommended load limits of 10% to 15% of a child’s body weight (BW) by many health professional associations [13]. Although these weight limits have been recommended in several states or countries, controversy continues to exist in the literature about the effects of backpack weight on back pain in children. Backpack loads should be reduced both because they exceed proportionally the limits set for adults [14] and frequently cause discomfort, and because fatigue during backpack carrying and time spent bearing the backpack on the shoulders are parameters associated with back pain.

In Uganda, literature search has revealed no previous study on back pain among school going children and there is no data available describing the current use of schoolbags by pupils in the country, especially among day scholars who commute to school every day. This study was set to investigate the prevalence of back pain and its association with schoolbag use in pupils.

## Methods

This was a cross-sectional descriptive study carried out among school children from six primary schools. Ethical approval was obtained from the Makerere University School of Medicine research and ethics committee. The sample size was calculated using the online OpenEpi software for sample size calculation for proportions (http://www.openepi.com) [15]. This was obtained using the assumption that each of the 6 schools had 4 streams of 100 pupils each giving a sample population of 2400 pupils in the candidate classes. We used an assumed prevalence of LBP of 50% as was reported in the study by Haselgrove et al., 2008 [16] and precision of 5% (delta). To allow for adequate power during sub analysis a confidence level of 99% for (1-β) was selected to give a sample size of 520 pupils. To this a 50% additional allowance for non- response or refusal to consent on the part of the pupils parents. This gave a final targeted sample size of 780 pupils. These pupils had to assent before recruitment in the study. Schools located within Kampala the capital city of Uganda East Africa, were classified as urban and those from up-country districts were classified as rural.

Participants enrolled from five schools were in Primary seven while one school which was a private school contributed pupils of primary six because the P7 class had already gone on vacation after completing their final primary leaving examinations (PLE). The inclusion criteria were as follows: being in class primary seven or six, parental consent, pupil assent, ability to ambulate independently, and ability to wear a school bag while standing on a weighing scale. All children with pathological causes of back pain were excluded from the study. However, such children were given professional orthopaedic consultation and where necessary treatment was prescribed.

### Data collection

Only school children whose parents/guardians had given informed consent were eligible and of these only those who endorsed the assent forms were enrolled into the study. The pupils had their weights and heights measured. The weighing scales were placed on a flat surface in a corner of the classroom and set to zero. Children dressed in their uniforms with their shoes removed were then weighed using a digital weighing scale (AEG Electrolux ABS 408E, China) with 0.1 kg increments. The weight was first measured when carrying the school bag and then without the school bag and the difference between the two weights was recorded as the weight of the school bag. All values were documented on the questionnaire. The weighing scales were recalibrated after each measurement. Validated questionnaires were then administered and answered collectively as a group. One of the investigators would take the lead and guide the entire class through a series of question. Care was taken to simplify the questions as much as possible and explanations were given whenever questions arose. Some questions had to be translated into the respective local languages for ease of understanding whenever it was deemed necessary. The rest of the research team would circulate around the class to make sure that questionnaires were being filled fully and correctly. Questionnaires were then assessed for completeness before data entry.

### Study variables

The outcome variable was low back pain which was operationally defined as pain or discomfort in the low back region, from the lower rib curvature to the lower part of the seat region. Predictor variables included body weight, schoolbag weight, schoolbag weight as a percentage of body weight, type of schoolbag, how the bag was carried, pupil perception of bag weight and comfort while wearing the bag, and activities done after school.

### Statistical analyses

Questionnaire data was entered into a computer using Microsoft Excel and then imported into Stata 10.0. School bag weight as a percentage of body weight was computed by dividing the weight of the bag by the child’s weight. Responses were analyzed using frequency distributions and descriptive statistics. Chi-square cross tabulations were used to distinguish differences in response by location, perceptions of bag weight and comfort carrying bag, history of back pain, and carrying loads greater than 10% of body weight. Test for trend across ordered groups was used to analyze relationships between the mode of transport to school, time spent carrying the bags, time spent sitting after school and low back pain. Both linear and logistic regression analysis were performed to analyze the effects of schoolbag usage on back pain, and pupils’ perceptions on the weights of their bags. Results were considered significant when the p- value was less than 0.05.

## Results

A total of 783 consent forms were dispatched but only 532 parents (67.9%) consented for their children to be included in the study. These 532 pupils from 6 primary schools participated keeping the power (1-β) of the study above the calculated 99% for the targeted 520 pupils. The 532 school going pupils were from five public schools and one private school. They comprised of 237/532 (44.6%) males and 294/532 (55.4%) females. The mean age of the pupils was 13.6 years (range 10- 21 years ± 1.66). The mean age of pupils from the urban schools was 12.9 ± 1.9 compared with 14.3 ± 1.5 for the rural schools. The overall mean weight of the pupils was 46.82 kg ± 9.1(range 28.5- 84.9 kg) (males 46 ± 9.1, females 47.5± 9.1).

### School bag weight

The average schoolbag weight carried by the pupils was 3.78 kg ± 1.97 (range 0- 12.3 kg). The mean bag weight as a percentage of the body weight was 8.46% ± 5.0 (range 0- 31.3%). The weight of the schoolbags as a percentage of body weight as summarized in Table 1 showed younger pupils carrying heavier bags than the older ones. For this analysis the intercept for the regression line 1 (Table 1) corresponded to 10 year olds carrying bags whose weight was 13.3% of their body weight. This percentage of the school bag over the pupils’ body weight reduced by 1.3% for every additional year in the age of any two pupils. There were 164/532 pupils (30.8%) carrying bags which were more than 10% of their body weight and of these 122/164 (74.4%) pupils were from urban schools and 42/164 (25.6%) from rural schools. Urban pupils were carrying significantly heavier bags (mean 10.58% ± 5.29) than their rural counterparts (mean 6.59% ± 3.82) (*t (df* 530) = 10.04, p < 0.0001, *r* = 0.4).

**Table 1:** Regression equations

**Equation no:**	**Intercept**	**Coefficient**	**Standard error**
1	Percentage body weight	R squared =0.19	
	age	-1.297	0.119
	constant	13.283	0.478
2	Bag weight	R squared change = 0.150	
	location	-1.527	
3	Bag weight	R squared change = 0.097	
	Age	-0.370	0.049
	constant	5.157	0.199

### School bag use

The most common type of bag carried by the pupils was the backpack (305/532, 57.3%), the other types of bags carried included shoulder bags, plastic bags and rucksacks. All pupils from the urban schools had school bags of which 79.9% were backpacks. For the rural pupils, only 102/278 (36.7%) had back packs while 43/278 (15.5%) of them had no school bags. Of the few pupils who had bags with all the recommended safety features for comfortable bag carriage only 24/532 (4.5%) routinely used these features. For the backpacks, two hundred sixty seven (51.9%) used two shoulder straps to carry their bags while 146//509 (28.7%) used one shoulder strap. All pupils had scholastic materials in their bag with a few 150/531 (28.3%) having lunch packs and water bottles. Only 101/532 pupils (19%) had access to lockers which they used through-out the day.

The mode of transport and time spent carrying school bags to and from school are summarised in Table 2, indicating that about 77.7% of the pupils walked to and from school daily carrying their schoolbags, 43.9% were carrying their bags for more than 20 minutes on their way to school whereas 54.7% had to carry their bags for the same duration on their way back home from school especially in the rural schools. There was a significant difference in the duration of school bag carriage between urban and rural children (Test for trend across ordered groups z = 6.95, p < 0.0001) however the effect was medium *(r =* 0.30).

**Table 2:** Mode of transport and time spent carrying the school bag

***Mode of transport***	***Urban (n = 251)***	***Rural (276)***
Walking	137 (54.6)	240 (87)
Bicycle	1 (0.4)	9 (3.3)
Car	64 (25.5)	7 (2.5)
Taxi/ Bus	36 (14.3)	18 (6.5)
Motorcycle	13 (5.2)	2 (0.7)
*Time spent carrying bag to school*	n = 252	n = 276
<5 minutes	71 (28.2)	37 (13.4)
5- 10 minutes	75 (29.8)	57 (20.7)
11- 20 minutes	34 (13.5)	22 (8)
21- 30 minutes	31 (12.3)	42 (15.2)
>30 minutes	41 (16.2)	118 (42.7)
Test for trend across ordered groups	z = 6.95, p < 0.0001.	
*Time spent carrying bag from school*	n = 252	n = 276
<5 minutes	67 (26.5)	30 (10.9)
5- 10 minutes	41 (16.3)	34 (12.3)
11- 20 minutes	37 (14.7)	29 (10.5)
21- 30 minutes	37 (14.7)	51 (18.5)
>30 minutes	70 (27.8)	132 (47.8)
	z = 5.76, p < 0.0001.	

### Musculoskeletal pain

Four hundred forty eight (448, 88.2%) of the pupils reported having had pain or discomfort in the body over the previous 2 weeks. Pain was greatest in the neck (24.5%), shoulders (42.1%), upper back (35.7%) and the lower back. Two hundred and one of pupils (37.8%) reported having had low back pain which had lasted for one day or longer with the male pupils being less significantly affected than the female pupils (odds ratio 0.54, 95% CI 0.37- 0.79). The pupils associated several activities with the causation of low back pain as shown in Table 3. About 35.4% of the children reported that carrying the schoolbag was the cause of body pain. Walking for long periods had a significant association with low back pain (odds ratio 2.67, 95% CI 1.38- 5.16). Logistic regression analysis showed that there was no significant difference in the odds of having back pain for the different types of school bag carried (odds ratio = 1.04 95% CI 0.864-1.252). Only 48 (9.3%) of the entire group had ever missed school because of back pain. However, among those with back pain 26.1% had ever missed school because of the pain. One hundred forty eight school children (28.6%) had ever had to rest or not play sports because of back pain while 36/520 children (6.9%) had ever been taken to the doctor because of back pain. Lower back pain in children using back packs and shoulder bags significantly differed between the urban and rural locations (z = 2.13, p = 0.03 and z = 2.33, p = 0.02 respectively). There was no significant relationship between the time spent carrying the bags on the way to school and back pain. There was also no significant difference in back pain between the urban and rural school children (Odds ratio 0.72, 95% CI 0.51- 1.03). There was no significant association between low back pain and schoolbag weight as a percentage of body weight (p = 0.37).

**Table 3:** Factors associated with low back pain; a comparison between urban

	***Urban(n = 253)***	***Rural (n = 278)***	***Odds ratio***	***95% CI***
Sitting for long	18 (7.1%)	26 (9.4%)	0.90	0.46- 1.74
Walking for long	33 (13)	16 (5.8)	2.67	1.38- 5.16
Carrying the bag	92 (36.4)	96 (34.5)	1.24	0.83- 1.85
Writing	1 (0.4)	3 (1.0)	0.43	0.04- 4.22
Sports/ playing	13 (5.1)	11 (4.0)	1.1	0.47- 2.56
Doing household chores	8 (3.2)	12 (4.3)	0.86	0.34- 2.20
No pain	88 (34.8)	114 (41)	1	
Comparison by sex	Male	Female		
	66 (32.8)	135 (67.2)	0.54	0.37- 0.79

After school hours the majority of pupils did household chores and studying. On the whole activities done after school did not have a significant effect on low back pain (z = -0.1, p = 0.92). Two hundred and twelve pupils (212, 40.5%) on average spent between 2- 4 hours seated every evening and this significantly affected the occurrence of low back pain (z = -3.063, p = 0.02 ).

### Perceptions of schoolbag weight

The majority of pupils reported that they felt their bags were either medium (242/532, 49.9%) or heavy (193/532, 39.8%). About half of them (49.8%) felt uncomfortable whenever they carried their bags and 119/305 (38.5%) did not like their bags because they were either oversized or heavy. There was a significant difference in the perception of the weight of the schoolbags between the rural and urban schools (Test for trend across ordered groups z = 5.36, p < 0.001). There was no significant difference in the percentage of the school bag weight compared to their body weight for pupils who perceived their bags as being light (mean 7.07% + 4.38) or medium (mean 8.14% ± 4.3) (*t* (290) = -1.6 *p* = 0.11). There was a significant difference in the percentage of the school bag weight compared to their body weight for pupils who perceived their bags as being medium (mean 8.14% ± 4.3) or heavy (mean 10.48% ± 5.47), *t* (*df* 358.22) = -4.85 *p* < 0.001). Urban pupils were less likely to complain about the weight of their bags being uncomfortable (odds ratio 0.362, 95% CI 0.249- 0.53).

## Discussion

The purpose of the study was to document low back and other musculoskeletal pains and describe their relationship with schoolbag use in pupils. Comparisons between pupils from rural and urban schools were also made. The prevalence of reported low back pain among pupils was 37.8% with males being less significantly affected than female pupils (odds ratio 0.54, 95% CI 0.37- 0.79). This prevalence rate is consistent with rates reported elsewhere [8-11,17], in some studies it has been reported to be as high as 65% [7]. In addition to low back pain children also experienced pain in the upper body involving the neck, shoulders and upper back. Pain in these areas is associated with carrying heavy loads [5,16]. Carrying a heavy school bag for long periods of time could result in repetitive stress injuries to the growing body. This follows the shifting of the child’s centre of gravity in the direction of the load when carrying a backpack [5,18]. To compensate, the child will typically leans in a direction opposite to the force. For example, to compensate for a heavy backpack worn low over the sacrum, the individual typically moves the head and trunk forward.

Another common strategy is lumbar hyperextension accompanied by hand support on the shoulder straps. Such postural deviations can hamper the natural shock absorption abilities of the spine and require greater muscle activity to prevent the individual from falling as a result of the increased forces and moments about the spine. These heavy school bags result in several postural changes at the head and trunk placing soft tissues at a biomechanical disadvantage resulting in fatigue and injury. The mean schoolbag weight as a percentage of body weight was 8.5% however about 30.8% of the children had bags which were more than the recommended limit of 10% with the urban school children carrying heavier bags. About 35.4% of the pupils reported that they developed pain whenever they carried their school bags however just like in other studies schoolbag weight was not associated with low back pain (p = 0.37) [19]. About 30.8% of pupils had bags which weighed in excess of 10% of their body weight, this was consistent with other studies which report rates between 15%- 50% [14,20-23]. Researchers have explored whether there is a critical backpack weight-to body ratio that if exceeded affects health. Backpack loads exceeding 10% of body weight have been shown to increase energy consumption, [24] increase trunk forward lean, [5,25] and result in decreased lung volumes [26]. These three factors lead to reduced oxygen partial pressure (PO2) resulting in anaerobic respiration and eventual fatigue. There are several reasons for heavier backpack loads among school going children, these include: pressure to attain higher academic performance leading to children getting more homework. Whereas the contents of the schoolbags were scholastic in nature, urban school children carried more text books and hard cover counter books as compared to the conventional lighter exercise books carried by their rural counterparts, in addition, urban children also had lunch packs/ water bottles as additional contributors to the school bag weight. To prevent low back pain and associated muscle fatigue, muscle strain, and other serious back injuries, many experts recommend limiting the school bag load to 10% to 15% of body weight [13,14].

The duration of time spent by the children carrying their school bags had a significant positive correlation with the occurrence of reported lower back pain (r = 0.30, p < 0.0001). This is of concern if one considers that with bigger capacity backpacks a pupil can accommodate several more items as compared to the other bag types.

Experts recommend wide, padded shoulder straps for comfort and greater distribution of weight across the shoulders, padded back for comfort and protection, and multiple compartments for distribution of load [27]. Many of the backpacks found with the participating pupils did not have these recommended ergonomic features to protect the back. Among the few who had backpacks with all the recommended features only 24 (4.5%) routinely used them. These features stabilize and better distribute the load in the backpack and increase comfort. They are, however, more costly and less commonly used by children of school age. Many backpacks commonly used by children feature adjustable straps to allow varied placement of the backpack on the user’s back. School children should be encouraged to use two shoulder traps and also be taught the proper use and importance of the several features on their bags since the majority of them have to walk in excess of 20 minutes daily to and from school. To further reduce on the effects of bag weight schools ought to provide students with lockers for storage of their scholastic materials. Schools should also have fully functional libraries where students can sit, read and borrow text books instead of carrying them daily in their bags. Sadly though, only 22.2% of the pupils had access to lockers, and no school had a fully functional library because of overcrowding. This resulted in the reported carrying of schoolbags between classes for fear of theft or loss of property.

There was a significant correlation between the time pupils spent sitting after school and the occurrence of back pain. Several children reported sitting for more than 6 hours at night studying! Parents should limit the amount of time their children spend reading at night. A study on low back pain showed that children who watch a small amount of television per day (<1 hour) were at no greater or lesser risk of back pain than those who watched none, [8] whereas watching 1–2 hours, and watching >2 hours were associated with a 70% and a 210% increase in the odds of LBP, respectively. Other studies have shown similar results [28].

The study also investigated pupils’ subjective perceptions of their daily schoolbag loads. The majority of the pupils reported that they felt their bags were either medium (49.9%) or heavy (39.8%). About half of them (49.8%) felt uncomfortable whenever they carried their bags, several of them did not like their bags because they were either oversized or heavy. These figures are comparable to another study which reported a perception of fatigue and schoolbag heaviness of 50% [16]. Younger city children were carrying heavier bags and therefore are more prone to spinal damage when compared to their colleagues in rural settings. To decrease injury and improve comfort, experts recommend that children use backpacks that match the size of the child [13]. There was a significant difference in these perceptions between rural and urban schoolchildren with those from the urban schools feeling significantly more uncomfortable. Location change from city to non-city/rural settings led to a 3.86% reduction in the percentage of bag weight compared to body weight. This means that the urban children are carrying significantly heavier bags than the rural children and that when a child complains that a previously light bag has now become heavy this should be taken seriously. It is of interest to note that the rural children were twice as likely to complain that they found the weights of their bags uncomfortable as compared to urban children. In view of the recommended cut off limits at 10% it can be seen that a child in an urban location is more exposed to spinal damage given that they are younger (Table 1, equation 3), carrying heavier bags (Table 1 equation 2) and are less likely to complain about the weight of their bags (odds ratio 0.362, 95% CI 0.249- 0.53). Physicians involved in the treatment of children know, from direct experience, that backpack carrying is perceived by parents as a social problem, it is high time this notion changed!

## Conclusions

Research on back pain in children is difficult because the aetiology may be multifactorial. The majority of pupils have musculoskeletal pain especially in the neck shoulders and lower back. Pupils carry heavy schoolbags with a significant proportion of them carrying school bags of more than 10% of their body weight. There were multiple factors of school bag usage that were associated with lower back pain. School children, schools and families are equally involved in determining the weight of schoolbags, and all could contribute to reducing it. Parents remain the best advocates for safety promotion and should represent the group most likely to help to significantly reduce the number of schoolbag related injuries by checking backpack weights and contents. Studies have shown that parents seldom check the weight and contents of children’s schoolbags [29]. A second line of prevention should focus within the school. If children are provided with lockers in which to keep their school bags and other scholastic materials while at school a number of potential repetitive strain injuries may be averted. Currently, many professional organizations are communicating virtually the same message: choose the right size backpack; pack well and empty out unnecessary items; wear straps on both shoulders; and carry less than 10%- 15% of body weight.

## Competing interests

We declare that there are no competing interests.

## Authors’ contributions

ESM and IGM conceived and designed the study. All contributed in the data collection. ESM, IGM and WB analyzed the data. JO, JK and EM prepared the manuscript, final proof reading and approval of the manuscript was done by OJ. All authors read and approved the final manuscript.
